# Aberrant Modulations of Neurocognitive Network Dynamics in Migraine Comorbid With Tinnitus

**DOI:** 10.3389/fnagi.2022.913191

**Published:** 2022-06-22

**Authors:** Liping Lan, Yin Liu, Jin-Jing Xu, Di Ma, Xindao Yin, Yuanqing Wu, Yu-Chen Chen, Yuexin Cai

**Affiliations:** ^1^Department of Otolaryngology, Sun Yat-sen Memorial Hospital, Sun Yat-sen University, Guangzhou, China; ^2^Department of Radiology, Nanjing First Hospital, Nanjing Medical University, Nanjing, China; ^3^Department of Otolaryngology, Nanjing First Hospital, Nanjing Medical University, Nanjing, China; ^4^College of Information Science and Technology, Nanjing Forestry University, Nanjing, China

**Keywords:** migraine, tinnitus, neurocognitive network, executive control network, fMRI

## Abstract

**Purpose:**

The possible relationship between migraine and tinnitus still remains elusive although migraine is often accompanied by chronic tinnitus. Several neuroimaging studies have reinforced the cognitive network abnormality in migraine and probably as well as tinnitus. The present work aims to investigate the dynamic neurocognitive network alterations of migraine comorbid with tinnitus.

**Materials and Methods:**

Participants included migraine patients (*n* = 32), tinnitus patients (*n* = 20), migraine with tinnitus (*n* = 27), and healthy controls (*n* = 47), matched for age and gender. Resting-state functional magnetic resonance imaging (rs-fMRI) with independent component analysis (ICA), sliding window cross-correlation, and clustering state analysis was used to detect the dynamic functional network connectivity (dFNC) of each group. Correlation analyses illustrated the association between clinical symptoms and abnormal dFNC in migraine as well as tinnitus.

**Results:**

Compared with healthy controls, migraine patients exhibited decreased cerebellar network and visual network (CN-VN) connectivity in State 2; migraine with tinnitus patients showed not only decreased CN-VN connectivity in State 2 but also decreased cerebellar network and executive control network (CN-ECN) connectivity in State 2 and increased cerebellar network and somatomotor network (SMN-VN) connectivity in State 1. The abnormal cerebellum dFNC with the executive control network (CN-ECN) was negatively correlated with headache frequency of migraine (rho = −0.776, *p* = 0.005).

**Conclusion:**

Brain network characteristics of migraine with tinnitus patients may indicate different mechanisms for migraine and tinnitus. Our results demonstrated a transient pathologic state with atypical cerebellar-cortical connectivity in migraine with tinnitus patients, which might be used to identify the neuro-pathophysiological mechanisms in migraine accompanied by tinnitus.

## Introduction

Migraine, a neurological disorder, presents with attacks of throbbing headache and neurological symptoms including vomiting, nausea, hypersensitivity to environmental stimuli, and mood changes (Bigal and Lipton, 2008). The development and course of migraine vary with each patient, where a subset of patients gets worsen over a period of months or years in form of an increased frequency of attacks. Tinnitus is a phantom sound perception in the absence of external stimuli with a prevalence of 12–30% worldwide (Shargorodsky et al., 2010; Piccirillo et al., 2020). It is worth noting that migraine or headache troubles nearly 26–47% of those with tinnitus (De Ridder et al., 2015), which has attracted many researchers to investigate the association between tinnitus and migraine recently. Thus, migraine, regarded as a risk factor, may dramatically decrease quality of life coupled with tinnitus. It has been suggested that migraine and tinnitus share a common neuropathological circuit, reflected in the similar disrupted thalamocortical activity (De Tommaso et al., 2014; De Ridder et al., 2015; Xu et al., 2021). Nevertheless, though migraine is often accompanied by chronic tinnitus, their potential pathophysiological relationship remains vague.

Brain networks dynamically and rapidly reorganize and coordinate on subsequent temporal scales to allow the execution of neurocognitive processes in a timely fashion (Bressler and Tognoli, 2006; Li et al., 2020) and this has been also proposed for tinnitus (De Ridder et al., 2014). Tinnitus is a complicated brain disorder usually suffering from cognitive and emotional symptoms and involves reorganization of brain networks’ memory, mediating perception, distress, salience, and attention (Chen et al., 2017a,c). Tinnitus has been characterized by aberrant intra- or inter-connectivity in large-scale brain networks (Kam et al., 2020). For example, the frontoparietal network (FPN) is involved in the top-down regulation of attention and emotion, which may explain deficits in tinnitus (Sedley et al., 2016).

Several studies using functional MRI (fMRI) have reinforced the brain network abnormality in tinnitus and probably also migraine, including alterations in auditory and extra-auditory distributed cortical networks (Llinás et al., 1999; Roberts et al., 2010; De Ridder et al., 2015; Hayes et al., 2021). Increased connectivity between auditory and limbic network have been observed (Schlee et al., 2008; Kim et al., 2012; Maudoux et al., 2012a,b). Da Silva et al. found enhanced cortical thickness in the somatosensory cortex (SSC) as well as in the visual-motion processing regions in patients with migraine with and without aura (Granziera et al., 2006; DaSilva et al., 2007), suggesting that migraine attacks may lead to neuro-plastic changes in the SSC where the head and the face are somatotopically represented. Moreover, functional connectivity (FC) analyses have explored the functional organization of specific brain networks responsible for sensory processing (Sprenger and Borsook, 2012; Schwedt et al., 2015). The aberrant visual network has been reported in patients with migraine (Hadjikhani et al., 2001; Granziera et al., 2006; Palm-Meinders et al., 2017; Wang et al., 2017; Zhang et al., 2017b; Gaist et al., 2018). Recently, Tedeschi et al. (2016) found lingual gyrus, as one of the main components of the visual network had stronger functional connectivity in patients with migraine with aura (MWA) but its structure or microstructure remained normal compared with healthy controls and individuals with migraine without aura (MWoA). Moreover, altered remote FC to higher-order networks has been detected in the centro-parietal regions involving sensorimotor networks (Zhang et al., 2017a; Chong et al., 2019). This may hint that sparse long-range network connectivity to higher-order regions and aberrant network activity in centro-parietal sensorimotor regions could commonly generate the neuro-pathophysiological characteristics of response inhibition in patients with MwoA.

In line with the theory that migraine is actually an altered neurocognitive cortical process, Chen et al. (2020) found the normal regulation of prepotent responses might be destructed by cortical disexcitability of the prefrontal executive network and centro-parietal sensorimotor network in migraineurs. Response inhibition was abnormal probably caused by the weaker neural activities within the prefrontal executive networks in patients MwoA. Therefore, it was considered a vital element of the executive system (Logue and Gould, 2014; Jahanshahi et al., 2015). Other relevant studies also have perceived the looser functional connection in the prefrontal executive network including the middle frontal gyrus as well as the dorsal anterior cingulate cortex (Russo et al., 2012; Chong et al., 2019; Filippi and Messina, 2019).

The cerebellum is correlated with pain sense (Moulton et al., 2010) and has been proved to cause migraine. Russo et al. (2019) found a significantly increased activation in cerebellar cortices both in patients with MWA and MwoA after thermal stimulation of the trigeminal nerve. Anatomically, the gray matter volume of the cerebellum was increased in response to trigeminal pain, and functionally, the neural response in this region was regulated by the stage and severity of migraine (Mehnert and May, 2019). The relevant regions including the primary motor cortex, dorsolateral prefrontal cortex, periaqueductal gray, inferior parietal lobule, primary somatosensory cortex, and parahippocampal gyrus were considered to participate in cognitive, sensorimotor, emotional, and pain information processing leading to migraine (Moulton et al., 2010; Mehnert and May, 2019; Russo et al., 2019). The visual pathway may also interact with the pain perception regulatory network in view of increased functional connectivity between the left cerebellum and lateral geniculate body.

Therefore, the present work aims to investigate the dynamic neurocognitive network alterations of migraine and tinnitus and whether clinical features are associated with such abnormalities. We used to construct brain functional networks of patients with migraine, chronic tinnitus, migraine with tinnitus, and healthy controls, and analyzed the dynamic functional network connectivity (dFNC) alterations using independent component analysis (ICA), sliding window cross-correlation, and clustering state analysis. Static functional network connectivity (sFNC) can be applied to assess the temporal correlation between brain regions over the whole period of fMRI acquisition; however, their applicability is restricted by oversimplified analysis excluding temporal dynamics (Allen et al., 2014). Important details on neurological diseases that might not be accessible through static connectivity can be obtained through dFNC (Sakoğlu et al., 2010). By exploring the neurocognitive network characteristics of migraine with tinnitus, possible neuropathological mechanisms of migraine comorbid with chronic tinnitus may be tested. We assumed that abnormal dFNC alterations would be detected within some specific networks in patients with migraine along with tinnitus.

## Materials and Methods

### Participants

This study was approved by the Research Ethics Committee of the Nanjing First Hospital. All the participants provided written informed consent. This study included four groups of participants, which are the following: (1) patients with migraine, (2) patients with chronic tinnitus, (3) patients with migraine with tinnitus, and (4) healthy controls.

According to the International Classification of Headache Disorders, Third Edition (beta version) (ICHD-3 beta), 32 episodic migraineurs without aura were recruited from the Department of Neurology in our hospital, containing 22 with unilateral headache (right/left: 10/12) and 10 with a bilateral headache or no side preference. All subjects were right-handed, none had chronic neurologic or psychiatric conditions, and none took daily medications other than vitamins or oral contraceptives. No subjects used analgesics for any reason more than 8 days per month. No subject was taking a migraine preventive medication. Duration of migraine was recorded as well as attack frequency.

A total of forty-seven patients with chronic bilateral tinnitus (duration > 6 months) were recruited from the Department of Otolaryngology in our hospital. The pure tone audiometry (PTA) examination, as well as the Iowa version of the Tinnitus Handicap Questionnaires (THQ) (Kuk et al., 1990), was applied to evaluate the hearing threshold, tinnitus severity, and tinnitus distress. Any individuals whose PTA thresholds were ≥25 dB HL at the frequencies of 0.25, 0.5, 1, 2, 4, and 8 kHz (defined as hearing loss) were excluded from our research.

Furthermore, 47 patients with chronic tinnitus were divided into the migraine group (27 individuals) and the non-migraine group (20 individuals), respectively. In the migraine group, 8 had a right-side unilateral headaches, 11 had left-side and 8 had bilateral or no side preferential headaches, respectively. Moreover, 47 healthy control subjects were included in this study. None of these subjects was known to suffer from chronic tinnitus or migraine and were group matched for age, gender, and education. The excluded criteria were as follows: individuals had (1) hyperacusis, pulsatile tinnitus, and Meniere’s diseases; (2) head injury, anemia, stroke, Alzheimer’s disease, major depression, and other neuropsychiatric diseases; (3) MRI contraindications; (4) thyroid dysfunction, cancer, damaged liver/kidney function, and other organic diseases.

### Magnetic Resonance Imaging Acquisition

A 3T Philips Ingenia scanner (Philips Medical Systems, Best, Netherlands) with an eight-channel phased-array head coil was applied to obtain all resting-state image data. During scanning, subjects should lie tranquility keeping their eyes closed, but not fall asleep or think about anything peculiar. Also, any head movement was not allowed in this process. We used the earplugs (Hearos Ultimate Softness Series, New York, NY, United States) that could attenuate scanner noise by approximately 32 dB. After an 8-min and 10-s scanning, resting-state functional imaging data was acquired by a gradient echo-planar imaging sequence with the following specifications: repetition time (TR)/echo time (TE) = 2,000/30 ms; slices = 36; thickness = 4 mm; gap = 0 mm; field of view (FOV) = 240 mm × 240 mm; acquisition matrix = 64 × 64; and flip angle (FA) = 90°. While structural 3D T1-weighted images were obtained by the 3D turbo fast-echo T1WI sequence (TR/TE = 8.1/3.7 ms; slices = 170; FA = 8°; thickness = 1 mm; gap = 0 mm; FOV = 256 mm × 256 mm; and acquisition matrix = 256 × 256).

### Magnetic Resonance Imaging Data Preprocessing

Using the SPM12 software^1^ implemented in MATLAB (version R2016b, MathWorks, Natick, MA, United States), we performed the resting-state fMRI data preprocessing. Firstly, the first 10 scans were deleted to allow for the steady-state of magnetization and the patient’s adaptation to the scanning environment. Secondly, the inter-scan head motions were corrected by the realignment to the first volume. Thirdly, according to the tissue probability maps, it was divided into gray matter, cerebral spinal fluid, and white matter. Fourthly, non-linear transforming was used for the normalization into the standard Montreal Neurological Institute template while spatial smoothing was performed by 6-mm full width at half-maximum Gaussian kernel.

### Group Independent Component Analysis

After data preprocessing, we used independent component analysis (ICA) analysis to extract the spatial ICs and identify resting-state networks (RSNs) from the data of all subjects in the group ICA function of the fMRI Toolbox (GIFT) (Calhoun et al., 2001; Erhardt et al., 2011). Firstly, the data reduction was followed by principal component analysis, which evaluated the ICA according to the aggregate data of the subjects (Li et al., 2007). The number of ICs was evaluated by the minimum description length (MDL) criteria. Then, using the InfoMax algorithm building in the GIFT performed the proper ICA. Finally, the value of connectivity intensity within each IC was transformed into Z-score for showing the degree of correlation between a given voxel and its corresponding components in the time series (Calhoun et al., 2001). Based on previous rs-fMRI studies, 11 independent components were finally identified as RSNs by visual inspection among the results of ICA (30 ICs).

### Dynamic Functional Network Connectivity Analysis

In order to compute the dFNC between ICA time processes, a sliding time-window method was applied to compute the dFNC among ICA time courses was calculated by the sliding time-window approach, where the window was set at 20-TRs width convolved with a Gaussian (σ = 3 TRs) and each step length was 1 TR (Du et al., 2016). Therefore, each individual’s FNC data was segmented into 128 windowed FNC. Based on the method proposed in an earlier study (Damaraju et al., 2014), the inter-component covariance was calculated. The windowed covariance matrices (component × component × window) in the time series reflected the altered features of FNC in each individual.

All the dynamic FNC windows were eventually allocated into two clusters by the K-means clustering algorithm, which was calculated with 500 iterations and 150 repeats of dFNC windows in the squared Euclidean distance method (Malhi et al., 2019). The center of clustering can be thought of as the average patterns that participants tend to return to during the experiment (Miller et al., 2016). Based on the elbow criterion, defined as the ratio of intra- to inter-cluster distances, the algorithm was dedicated to matching the optimal value of k (minimized k-value) and finally evaluated the targeted value of k is 2 in the search window k is 2–10 (Damaraju et al., 2014). We evaluated the differences in the characteristics of each dFNC state between groups on the group level of dFNC states.

The differences of each dFNC state between the two groups were calculated by an independent two-sample *t*-test corrected for false discovery rate (FDR), where *p* < 0.05 was considered significant. The characteristic parameters of dFNC states including reoccurrence fraction, mean dwell time, and the number of transitions were also investigated. Meanwhile, the comparison of these parameters was conducted by an independent two-sample *t*-test (*p* < 0.05, FDR corrected). Mean dwell time is defined as how long the subjective stay in a certain state. The reoccurrence fraction is calculated as the proportion of the total number of windows belonging to a certain state, and the number of transitions is the number of changes from one state to another, representing the stability of FNC over time.

### Statistical Analysis

All statistical analysis was calculated by IBM SPSS 25 (IBM Corporation, Armonk, NY, United States). The differences in demographic and clinical information between the two groups were evaluated by the Chi-square tests (for categorical variables) and the independent two-sample *t*-test (for continuous variables). The correlations between the clinical characteristic and dFNC attributes, such as reoccurrence fraction, mean dwell time, and the number of transitions were calculated by Spearman’s correlation analysis and controlled for the variable including age and gender. The statistical significance was set at *p* < 0.05.

## Results

### Demographic Characteristics

**FIGURE 1:**
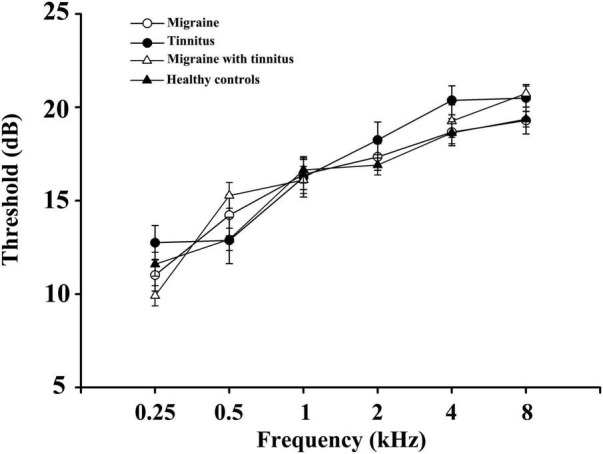
Average hearing thresholds of migraine patients, tinnitus patients, migraine patient with tinnitus, and healthy controls. Data are presented as mean ± SD.

As Table 1 demonstrated, no significant differences were found in age, gender, disease duration, hearing thresholds, THQ scores, and VAS scores between the four groups (patients with migraine, chronic tinnitus, migraine with tinnitus, and healthy controls). However, patients with migraine along with tinnitus had worse HIT6 scores and higher headache frequency than migraine patients (*p* < 0.05). There were no significant differences in hearing thresholds between the four groups (Figure 1).

**TABLE 1:** Demographics and clinical characteristics.

	Migraine (*n* = 32)	Tinnitus (*n* = 20)	Migraine with tinnitus (*n* = 27)	Healthy controls (*n* = 47)	*P*-value
Age (year)	37.91 ± 9.38	40.55 ± 10.42	37.78 ± 7.24	41.91 ± 8.73	0.136^a^
Gender (male/female)	5/27	8/12	5/22	11/36	0.207^c^
Mean HT (dB)	16.14 ± 2.38	16.75 ± 2.50	16.94 ± 1.44	16.22 ± 2.18	0.397^a^
Disease duration (months)	46.50 ± 28.06	–	46.67 ± 28.89	–	0.982^b^
Headache frequency	4.00 ± 1.83	–	9.30 ± 3.00	–	<0.001^b^
HIT-6 score	57.28 ± 9.34	–	62.37 ± 2.98	–	0.006^b^
VAS score	6.47 ± 1.46	–	5.85 ± 1.03	–	0.063^b^
THQ score	–	48.12 ± 14.76	50.62 ± 14.31	–	0.562^b^

*Data were presented as Mean ± SD. P-value < 0.05 were considered statistically significant.*

*^a^One-way ANOVA.*

*^b^Two-sample t-tests.*

*^c^Chi-square test.*

*Abbreviations HIT-6, Headache Impact Test-6; VAS, visual analog scale; THQ, Tinnitus Handicap Questionnaires; HT, Hearing thresholds.*

### Resting-State Networks

**FIGURE 2:**
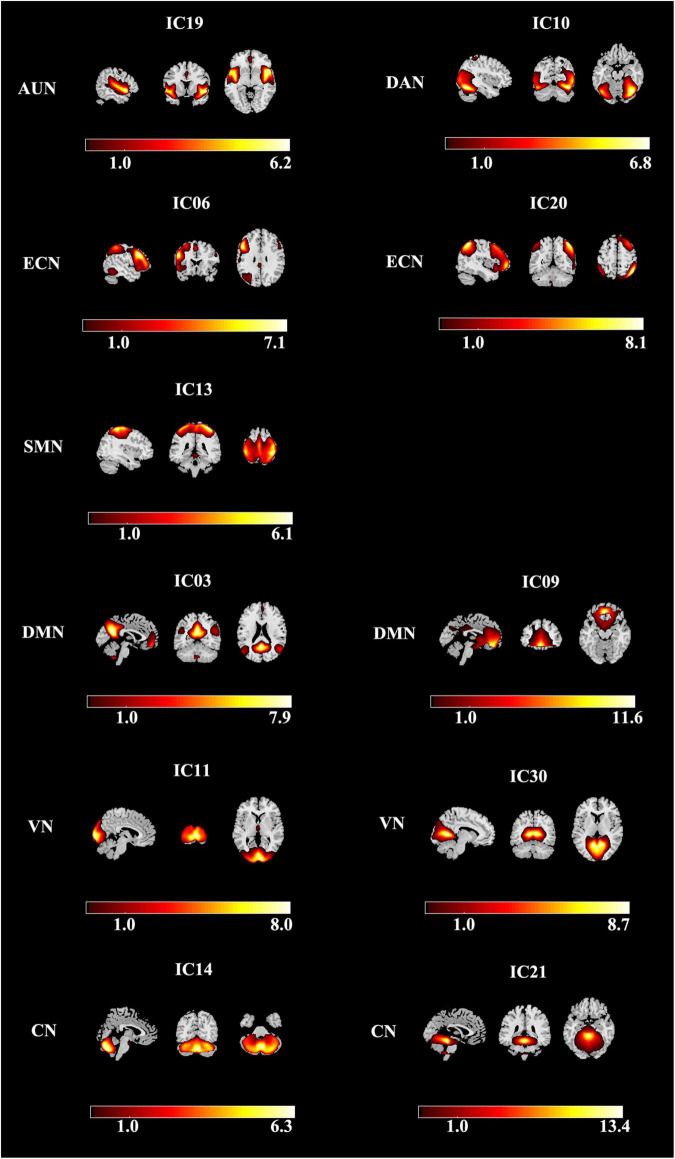
Spatial maps of identified resting-state networks are divided into seven different functional domains, namely, AUN, DAN, ECN, SMN, DMN, VN, and CN. AUN, auditory network; DAN, dorsal attention network; ECN, executive control network; SMN, sensorimotor network; DMN, default mode network; VN, visual network; CN, cerebellum network.

Using group ICA, seven meaningful RSNs were identified (Figure 2): the auditory network (AUN; IC19) primarily included bilateral middle, superior temporal gyrus, and insular. The dorsal attention network (DAN; IC10) mainly consists of the precentral and superior frontal cortex with the orbital part, ventral precentral, middle frontal gyrus, and bilaterally the intraparietal sulcus. The executive control network (ECN; IC06 + 20) included several medial frontal areas, containing the paracingulate and the anterior cingulate. The sensorimotor network (SMN; IC13) includes the bilateral precentral, medial, and postcentral frontal gyrus and the supplementary motor area. The default-mode network (DMN; IC03 + 09) primarily included the bilateral inferior parietal gyrus, posterior cingulate/precuneus, superior frontal gyrus, medial frontal gyrus, and angular gyrus. The visual network (VN; IC11 + 30) involved the middle and superior occipital gyrus, fusiform gyrus, and the temporal-occipital regions. And cerebellum network (CN; IC14 + 21) is located in bilateral cerebellum hemispheres.

### Group Difference of Occurrences and Dynamic Functional Network Connectivity Patterns

**FIGURE 3:**
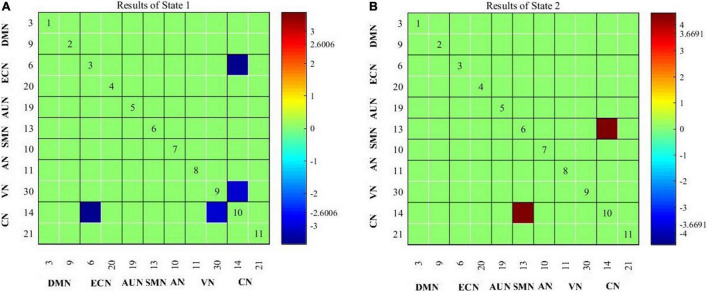
Connectivity results for State 1 **(A)** and State 2 **(B)** evaluated using two-sample *t*-tests, and the significance was corrected using false discovery rate (FDR).

Compared with healthy controls, patients with migraine exhibited decreased cerebellar network and visual network (CN-VN) connectivity in State 1; compared with healthy controls, patients with migraine along with tinnitus showed not only decreased CN-VN connectivity in State 1 but also decreased cerebellar network and executive control network (CN-ECN) connectivity in State 1 (Figure 3A) and increased cerebellar network and somatomotor network (SMN-VN) connectivity in State 2 (Figure 3B).

### Correlation Between Cerebellar-Cortical Dynamic Functional Network Connectivity and Clinical Traits

**FIGURE 4:**
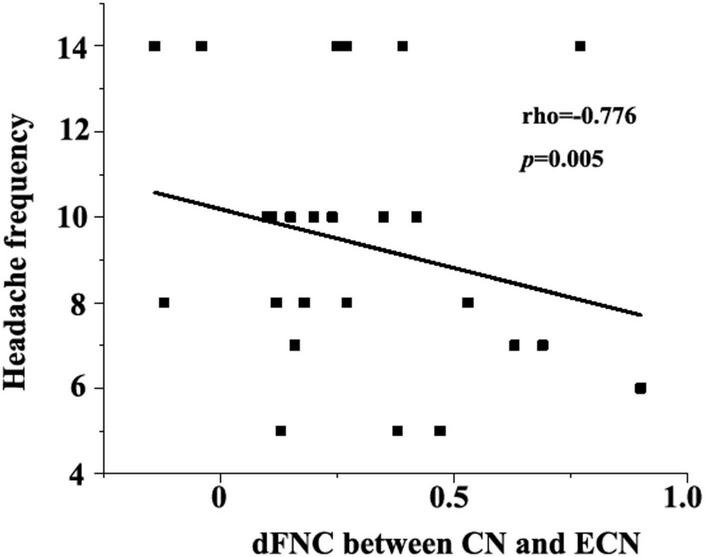
Negative correlation between dFNC (CN-ECN) and headache frequency (rho = −0.776, *p* = 0.005).

As shown in Figure 4, the abnormal cerebellum dFNC with the executive control network (CN-ECN) was negatively correlated with headache frequency of migraine (rho = −0.776, *p* = 0.005). Besides, the association between the clinical traits with the FNC measures was analyzed and no other significant correlations were observed.

## Discussion

In the present study, we identified two reoccurring dFNC states that exhibited significantly different connectivity patterns. The cerebellar network was highly synchronous in every state for patients with migraine. Positive dFNC in the cerebellar network with a somatomotor network (SMN-VN) was only observed in State 1. In-State 2, negative cerebellum dFNC connected with visual network, executive control network in patients with migraine. The abnormal cerebellum dFNC with the executive control network (CN-ECN) was negatively correlated with headache frequency of migraine.

Researchers found abnormal interaction between the cerebellar network and visual network in migraineurs between attacks (Moulton et al., 2010). Such associations were directly revealed by our study. Furthermore, abnormalities in CN-ECN connectivity may disrupt habituation to external stimuli. Indeed, lack of habituation is a well-characterized aspect of migraine disease that may also account for hyperexcitability in migraineurs. Tinnitus and chronic pain can thus be conceptualized as a continuous and persistent prediction error (Moulton et al., 2010; Bauer et al., 2013).

We observed abnormal cerebellum dFNC in migraine compared with healthy controls. The cerebellum generally participates in pain and nociceptive processing (Vincent and Hadjikhani, 2007; Moulton et al., 2010). One previous study indicated that the cerebellar regions were activated in healthy subjects when seeing some unpleasant picture or stung by painful heat stimulation which suggested the potential relationship between cerebellum and pain stimulation and general aversive processing (Moulton et al., 2010). Meanwhile, activated cerebellar responses are able to be induced by aversive stimuli like noxious and negative emotional pictures (Mehnert et al., 2017). Another clue demonstrating an association between cerebellum and pain is that experimental pain sensation is abnormally altered after cerebellar infarction (Ruscheweyh et al., 2014). The hyperesthesia toward heat could lead to more apparent abnormalities on the side of the infarct (Ruscheweyh et al., 2014). The cerebellum was proved to participate in migraine based on previous relevant studies. Functionally, it was demonstrated that the neural activities in cerebellar regions were overactive in patients with MWA and MwoA when performed the thermal stimulation of the trigeminal nerve was (Russo et al., 2019). Structurally, the increase in the gray matter volume of the cerebellum was detected within migraineurs compared with healthy controls. A couple of cerebellar functions and structures might be regulated by the severity and stage of migraine (Mehnert and May, 2019). The above evidence suggested that the cerebellum was a crucial node within the migraine-related neural pathways. There is abundant descending afferent and ascending efferent neural connectivity between the cerebellum and cerebral cortex and subcortex, which are responsible for their information transmission and interaction within top-down and bottom-up pathways. These neural pathways, centered in the cerebellum, were proved to participate in multi-information processing, such as sensorimotor, cognition, pain, and emotion, which might promote the migraine generation (Moulton et al., 2010; Mehnert and May, 2019; Russo et al., 2019).

The cerebellum had increased functional connectivity to the visually related regions like the later geniculate nucleus, which hinted at the aberrant integration between the visual and pain perception network (Zhang et al., 2020). The abnormal central reorganization caused by chronic pain syndromes like migraine might weaken the anti-nociceptive ability of the brain, as reflected in reduced dynamic functional connectivity between the cerebellum and somatomotor network (Jia and Yu, 2017). The broken cerebellar inhibitory effect on trigeminal neuralgia might be induced by the looser connections between the thalamus and superior cortical regions (Mehnert and May, 2019). In patients with MwoA, the cerebellum relevant impaired functional connectivity widely existed in the whole-brain neural network involving the multi-sensory cortices and cognitive relevant regions (Qin et al., 2020). It has been demonstrated that the cerebellum participates in and regulates pain perception (Moulton et al., 2010). Moreover, previous studies also indicated the possible relationships between the cerebellum (paraflocculus) and the tinnitus mechanism (Chen et al., 2017b; Mennink et al., 2020). Therefore, we suggest that the cerebellar network may play a core role in patients with migraine along with tinnitus.

This finding is consistent with existing literature on the pathophysiologic basis of migraine. For instance, the visual cortex is hyperexcitable in interictal migraine for both migraines with and without aura. Further exploratory analyses demonstrated that migraineurs with and without photophobia did not differ in occurrence rates of dFNC states. A study (Datta et al., 2013) found that the activation in visual networks including the primary visual cortex and lateral geniculate in patients with MwA was stronger than in patients with MwoA and healthy controls. Furthermore, the visual hyperactivity and photophobia in patients with migraine might be caused by the aberrant neural sensitivity of the posterior thalamus, which is an important intermediate node for visual information transmission. The SMN, consisting of the primary motor cortex, premotor cortex, supplementary motor area, and primary somatosensory cortex (Zhang et al., 2017a), is a key network responsible for multipurpose high-order cognitive processing (Brennan and Pietrobon, 2018), which previously proved to participate in migraine. Altered neural activities in some SMN subregions may result from pain and cognition (Yu et al., 2012; Wang et al., 2016). Additionally, the migraine attack is a paroxysmal dysfunctional alteration disrupting afferent or efferent information modulating among multiple sensory systems (Xue et al., 2012). It has been repeatedly demonstrated that the subregions in SMN functionally interact with the central executive network (CEN) in patients with MwoA (Zhang et al., 2017a). The strength of functional connectivity within SMN was significantly associated with pain intensity and therapeutic effect in MwoA (Li et al., 2015).

Since the patients in our study were in the interictal stage, this finding may further support the specificity of this abnormal dFNC to the brain’s functional architecture in migraine. We also speculate that during or around an attack, these dFNCs may no longer be functioning similarly, and there may be differences between those with and without photophobia. In addition, although most patients in our study reported that they had phonophobia before the MRI scan, we did not find any abnormal auditory dFNC or any difference between occurrence rates between those with and without phonophobia.

There are several limitations in the present study. Firstly, the relatively small sample size may affect the statistical reliability of the present outcomes. Secondly, the features of migraine were just evaluated by the GAD-7, migraine frequency, and VAS scores in this study. Furthermore, a confounding factor related to the auditory system should be taken into account. Using earplugs during MR scanning seems not enough to completely avoid the disturbance from scanner noise, which probably affects the brain’s functional architecture.

## Conclusion

This study provided evidence that brain network characteristics of migraine with tinnitus patients may indicate different mechanisms for migraine and tinnitus. These findings suggest a transient pathologic state with atypical cerebellar-cortical connectivity in migraine with tinnitus patients, which may underlie the neurocognitive mechanisms of migraine comorbid with tinnitus.

## Data Availability Statement

The original contributions presented in the study are included in the article/supplementary material, further inquiries can be directed to the corresponding authors.

## Ethics Statement

The studies involving human participants were reviewed and approved by Research Ethics Committee of the Nanjing Medical University. The patients/participants provided their written informed consent to participate in this study.

## Author Contributions

LL and YL designed the study, performed the experiments, and wrote the manuscript. J-JX, DM, XY, and YW performed the experiments and analyzed the data. Y-CC and YC revised the manuscript. All authors read and approved the final manuscript.

## Conflict of Interest

The authors declare that the research was conducted in the absence of any commercial or financial relationships that could be construed as a potential conflict of interest.

## Publisher’s Note

All claims expressed in this article are solely those of the authors and do not necessarily represent those of their affiliated organizations, or those of the publisher, the editors and the reviewers. Any product that may be evaluated in this article, or claim that may be made by its manufacturer, is not guaranteed or endorsed by the publisher.

## Footnotes

1
http://www.fil.ion.ucl.ac.uk/spm/
